# Hypertension Is Associated With Intestinal Microbiota Dysbiosis and Inflammation in a Brazilian Population

**DOI:** 10.3389/fphar.2020.00258

**Published:** 2020-03-12

**Authors:** Gabriela Silveira-Nunes, Danielle Fernandes Durso, Luiz Roberto Alves de Oliveira Jr., Eloisa Helena Medeiros Cunha, Tatiani Uceli Maioli, Angélica Thomaz Vieira, Elaine Speziali, Rodrigo Corrêa-Oliveira, Olindo Assis Martins-Filho, Andrea Teixeira-Carvalho, Claudio Franceschi, Simone Rampelli, Silvia Turroni, Patrizia Brigidi, Ana Maria Caetano Faria

**Affiliations:** ^1^Departamento de Bioquímica e Imunologia, Instituto de Ciências Biológicas, Universidade Federal de Minas Gerais, Belo Horizonte, Brazil; ^2^Departamento de Medicina, Instituto de Ciências da Vida, Universidade Federal de Juiz de Fora – Campus Avançado de Governador Valadares, Governador Valadares, Brazil; ^3^Núcleo da Saúde, Universidade Vale do Rio Doce, Governador Valadares, Brazil; ^4^Departamento de Nutrição, Escola de Enfermagem, Universidade Federal de Minas Gerais, Belo Horizonte, Brazil; ^5^Fundação Oswaldo Cruz-FIOCRUZ, Instituto René Rachou, Grupo Integrado de Pesquisas em Biomarcadores, Belo Horizonte, Brazil; ^6^IRCCS Istituto delle Scienze Neurologiche di Bologna, Bologna, Italy; ^7^Department of Applied Mathematics, Institute of Information Technology, Mathematics and Mechanics (ITMM), Lobachevsky State University of Nizhny Novgorod, Nizhny Novgorod, Russia; ^8^Unit of Microbial Ecology of Health, Department of Pharmacy and Biotechnology, University of Bologna, Bologna, Italy

**Keywords:** gut microbiota, dysbiosis, hypertension, inflammation, immune profile

## Abstract

Hypertension is a major global health challenge, as it represents the main risk factor for stroke and cardiovascular disease. It is a multifactorial clinical condition characterized by high and sustained levels of blood pressure, likely resulting from a complex interplay of endogenous and environmental factors. The gut microbiota has been strongly supposed to be involved but its role in hypertension is still poorly understood. In an attempt to fill this gap, here we characterized the microbial composition of fecal samples from 48 hypertensive and 32 normotensive Brazilian individuals by next-generation sequencing of the 16S rRNA gene. In addition, the cytokine production of peripheral blood samples was investigated to build an immunological profile of these individuals. We identified a dysbiosis of the intestinal microbiota in hypertensive subjects, featured by reduced biodiversity and distinct bacterial signatures compared with the normotensive counterpart. Along with a reduction in Bacteroidetes members, hypertensive individuals were indeed mainly characterized by increased proportions of *Lactobacillus* and *Akkermansia* while decreased relative abundances of well-known butyrate-producing commensals, including *Roseburia* and *Faecalibacterium* within the *Lachnospiraceae* and *Ruminococcaceae* families. We also observed an inflamed immune profile in hypertensive individuals with an increase in TNF/IFN-γ ratio, and in TNF and IL-6 production when compared to normotensive ones. Our work provides the first evidence of association of hypertension with altered gut microbiota and inflammation in a Brazilian population. While lending support to the existence of potential microbial signatures of hypertension, likely to be robust to age and geography, our findings point to largely neglected bacteria as potential contributors to intestinal homeostasis loss and emphasize the high vulnerability of hypertensive individuals to inflammation-related disorders.

## Introduction

Hypertension is a major global health challenge, as it represents the main risk factor for stroke and cardiovascular disease, the number one cause of death worldwide, as well as for kidney disorders (Hillege et al., 2000; Mills et al., 2016; de Oliveira et al., 2017). In Brazil, high blood pressure (HBP) has reached 30% of the adult population, 5% in children and adolescents, and 50% in elderly people (Picon et al., 2012; Ministério da Saúde, 2013). The identification of the causes of hypertension is still challenging but it is widely accepted that its etiology is multifactorial, involving an intricate set of endogenous and environmental factors contributing to its onset and progression. To date, HBP values have indeed been associated with over 50 genetic loci and metabolic pathways (such as those involved in the renin–angiotensin–aldosterone system) (Levy et al., 2009, 2017; Newton-Cheh et al., 2009; International Consortium for Blood Pressure Genome-Wide Association Studies et al., 2011; Kato et al., 2011; Ganesh S. K. et al., 2013; Tragante et al., 2014; Menni et al., 2015; Xu et al., 2015; Liu et al., 2016), as well as with lifestyle habits, such as dietary salt intake, alcohol consumption and lack of physical activity (Fuchs et al., 2001; Karppanen and Mervaala, 2006).

The human gut microbiota, i.e., the large array of microbes inhabiting our gastrointestinal tract, plays key roles for our physiology, by producing a wide and diverse pool of bioactive small molecules, including short-chain fatty acids (SCFAs, mainly acetate, propionate, and butyrate), which strongly impact on metabolic homeostasis, and regulate immune and nervous system function (Turroni et al., 2018). Emerging evidence suggests a role for the gut microbiota in various disorders, at both enteric and systemic level, including cardiovascular disease (Gregory et al., 2015; Lynch and Pedersen, 2016; Li et al., 2017; Yan et al., 2017; Tang et al., 2019). Specifically, several studies in hypertensive rat models have directly or indirectly emphasized the relevance of the gut microbiota in the regulation of blood pressure (Khalesi et al., 2014; Gómez-Guzmán et al., 2015; Mell et al., 2015; Moghadamrad et al., 2015; Yang et al., 2015; Durgan et al., 2016). Only more recently, a few papers have explored the intestinal microbial alterations underlying hypertension, especially in cohorts of Chinese and United States adult individuals, providing a range of phylogenetic and functional signatures, and advancing a possible causal role of the gut microbiota dysbiosis in contributing to the pathogenesis of hypertension (Li et al., 2017, 2019; Yan et al., 2017; Dan et al., 2019; Sun et al., 2019).

In an attempt to further extend this knowledge, including other geographic locations and expanding the age range, here we characterized the fecal microbiota from 48 hypertensive and 32 normotensive Brazilian adult individuals, by next-generation sequencing of the 16S rRNA gene. In addition, the cytokine production of peripheral blood samples was investigated. While corroborating previous evidence on the decrease of health-promoting SCFA producers in hypertension, our findings unveil minority microbiota components potentially linked to HBP, along with an overall inflamed profile.

## Materials and Methods

### Study Population

Study population consisted of 80 volunteers aged >25 years, all resident in Governador Valadares, an urban municipality from the state of Minas Gerais (Southeast Brazil) and an endemic area for schistosomiasis. The subjects were participants of an institution called Casa Unimed, where they regularly went (twice a week) for exercise programs, lectures, community groups, music education, measurement of blood pressure and blood glucose tests, aimed at promoting health and preventing disease. Health conditions were checked according to standard clinical investigations and standard hematological and biochemical parameters. All subjects enrolled in this study were non-institutionalized and living in their own household.

The individuals were stratified into a control normotensive group and a hypertensive group based on the reported HBP (higher than 140 mmHg for systolic and 90 mmHg for diastolic) (Sociedade Brasileira de Cardiologia/Sociedade Brasileira de Hipertensão/Sociedade Brasileira de Nefrologia, 2010) taken previously at the time of diagnosis, and use of antihypertensive medication including diuretic drugs, such as hydrochlorothiazide and furosemide, angiotensin II receptor antagonists, such as losartan and valsartan, as well as adrenergic receptors antagonists (beta blockers), such as propranolol and atenolol. Some of the individuals used a combination of diuretics and other classes of drugs. The criterion for including them in the study was “reported and treated hypertension for more than 10 years,” which would characterize them as chronic patients with HBP. The control subjects were those with no report of HBP.

General exclusion criteria were: infections, acute or chronic inflammation, *Schistosoma mansoni* infection, autoimmune diseases, undernourishment, anemia, leucopoenia, mood disorders, neurodegenerative diseases, neoplasia, and use of hormones (steroids) and drugs in the previous 2 weeks (alcohol, antidepressants, immunosuppressants, anticoagulants, antibiotics). Written informed consent was obtained from each participant, prior to inclusion in our investigations. Fecal and blood samples were collected from consenting participants over a period of 2 weeks in February 2014. Blood samples were immediately processed for peripheral blood mononuclear cell (PBMC) separation as described below, and stored along with the fecal samples at −80°C until use. The fecal samples were used for both microbiota analysis and parasitological exams. *S. mansoni* infection was assessed by Kato–Katz parasitological method with quantitative *S. mansoni* egg counts, using two slides prepared from three stool samples. Twenty-four hour dietary recalls were conducted for each enrolled subject over 3 days. We used the standard method in nutritional science of sampling 2 week days and 1 weekend day in an attempt to fully account for dietary habits and fluctuations. Records were analyzed using TACO (Brazilian table of food composition) (Universidade Estadual De Campinas – Núcleo de Estudos e Pesquisas em Alimentac̨ão NEPA – UNICAMP, 2006), and the caloric contributions of the main macronutrient groups were calculated. This work was approved by the Ethical Committee of Universidade Federal de Minas Gerais (UFMG) as well as the National Research Ethics Committee (CONEP) of Brazil.

### Microbial DNA Extraction From Feces

Total microbial DNA was extracted using the QIAamp DNA Stool Mini Kit (QIAGEN, Hilden, Germany) with a modified protocol (Yu and Morrison, 2004). Briefly, 250 mg of feces were suspended in 1 ml of lysis buffer (500 mM NaCl, 50 mM Tris–HCl pH 8, 50 mM EDTA, 4% SDS). Four 3-mm glass beads and 0.5 g of 0.1-mm zirconia beads (BioSpec Products, Bartlesville, OK, United States) were added, and samples were treated in FastPrep (MP Biomedicals, Irvine, CA, United States) at 5.5 movements/sec for 1 min, repeated three times. Samples were heated at 95°C for 15 min, then centrifuged for 5 min at 14,000 rpm to pellet stool particles. Two hundred and sixty microliters of 10 M ammonium acetate were added to the supernatant, followed by 5-min incubation in ice and centrifugation at 14,000 rpm for 10 min. One volume of isopropanol was added to each sample and incubated in ice for 30 min. Precipitated nucleic acids were collected by centrifugation for 15 min at 14,000 rpm and washed with 70% ethanol. Pellets were suspended in 100 μl of TE buffer and treated with 2 μl of DNase-free RNase (10 mg/ml) at 37°C for 15 min. Protein removal by proteinase K treatment and DNA purification with QIAamp Mini Spin columns were performed following the manufacturer’s instructions. DNA concentration and quality were evaluated using NanoDrop ND-1000 spectrophotometer (NanoDrop Technologies, Wilmington, DE, United States).

### 16S rRNA Gene Sequencing and Data Processing

For each sample, the V3-V4 hypervariable region of the 16S rRNA gene was amplified using the 341F and 805R primers with added Illumina adapter overhang sequences as previously described (Turroni et al., 2017). The resulting amplicons of approximately 460 bp were cleaned up with Agencourt AMPure XP magnetic beads (Beckman Coulter, Brea, CA, United States). Indexed libraries were prepared by limited-cycle PCR using Nextera technology and further cleaned up as described above. Final libraries were pooled at equimolar concentrations, denatured with 0.2 N NaOH and diluted to 8 pM with a 20% PhiX control. Sequencing was performed on Illumina MiSeq platform using a 2 × 300 bp paired end protocol according to the manufacturer’s instructions (Illumina, San Diego, CA, United States).

Raw sequences were processed using a pipeline combining PANDAseq (Masella et al., 2012) and QIIME (Caporaso et al., 2010). Quality-filtered reads were binned into Operational Taxonomic Units (OTUs) at 97% similarity threshold using UCLUST (Edgar, 2010). Taxonomy was assigned using the RDP classifier against Greengenes database (May 2013 release). All singleton OTUs were discarded. Alpha rarefaction was performed using the Faith’s phylogenetic diversity, observed OTUs and Shannon index metrics. Beta diversity was estimated by computing weighted and unweighted UniFrac distances, which were used as input for Principal Coordinates Analysis (PCoA). OTUs assigned to genera of interest whose species were unclassified, were subjected to BLAST analysis (Altschul et al., 1990). Sequencing reads were deposited in the MG-RAST database (project ID, mgp84730). All statistical analysis was performed in R 3.3.2. UniFrac distances were plotted by the vegan package, and data separation in the PCoA was tested using a permutation test with pseudo-F ratios (function Adonis in vegan). Bacterial groups with the largest contribution to the ordination space were found by using the function envfit in vegan on the genus relative abundance and the weighted UniFrac ordination. Linear Discriminant Analysis Effect Size (LEfSe) algorithm with LDA score threshold of 2 (on a log10 scale) was applied after agglomerating data to genus and species level (Segata et al., 2011). Significant differences in alpha diversity and taxon relative abundances between groups were assessed by Mann–Whitney *U* test. When appropriate, *p* values were corrected for multiple comparisons using the Benjamini–Hochberg method. A *p* value ≤ 0.05 was considered as statistically significant, whereas a *p* value between 0.05 and 0.1 was considered a tendency.

### Cytokine Measurements

#### PBMC Obtainment

Peripheral blood mononuclear cells (PBMCs) were isolated by centrifugation (400 × *g* for 45 min at 18°C) over Ficoll-Hypaque gradient cushion (LMS Litton Biometries, Kensington, MD, United States), as described by Gazzinelli et al. (1983). After separation, PBMCs were washed in RPMI 1640 and resuspended to 1 × 10^7^ cells/ml. One hundred microliters of PBMC suspension, containing 1 × 10^6^ cells, were incubated for 6 days at 37°C, in humidified incubator containing 5% CO_2_, in 24-well flat bottom plates, in the presence of 800 μl of RPMI 1640 medium, supplemented with 5% AB Rh-positive heat-inactivated human serum (Sigma, St. Louis, MO, United States), containing 3% of antibiotic/antimycotic solution, from the stock mix containing 10,000 IU of penicillin, 10 mg of streptomycin, and 25 μg of amphotericin B per ml (Gibco-BRL, Grand Island, NY, United States). Afterward, the culture supernatants were collected and immediately frozen at −80°C, and kept stored until flow cytometric cytokine measurements.

#### Cytokine Beads Array

Quantitative cytokine analysis was performed using the Cytometric Bead Array method (Human Cytokine Flex Set, Becton Dickinson, Pharmingen, San Diego, CA, United States) for simultaneous measurement of IFN-γ, TNF-α, IL-17A, IL-6, IL-10, IL-4, and IL-2, as recommended by the manufacturer.

Twenty-five microliters of supernatant from PBMC cell cultures were incubated with a mixture of beads coated with capture antibodies specific for each cytokine, labeled with distinct red fluorescence intensities. After incubation and wash steps, the system was incubated with 17 μl of phycoerythrin (PE)-labeled anti-cytokine antibody. After washings and flow cytometric acquisition, the relative fluorescence intensity for each bead was determined, and the results expressed as pg/ml for each cytokine, according to the standard curves. A total of 400 events/cytokine-specific-bead were acquired on a FACSVerse^*rmTM*^ Bioanalyzer (Becton Dickinson). Data analysis was performed using the FCAP array software (Soft Flow, Inc., St. Louis Park, MN, United States).

#### Cytokine Data Analysis

In the present study, one of the strategies used was an innovative model to analyze the immune response, referred to as functional cytokine signature. This model was designed to convert quantitative cytokine measurements into a categorical analysis of low and high cytokine producers as previously proposed by Luiza-Silva et al. (2011). This approach is able to detect subtle differences not detectable by conventional statistical analysis. Briefly, this categorical approach first converts the continuous cytokine measurements expressed in pg/ml into categorical variables, referred to as “Low” or “High” cytokine levels, taking the global median value as a specific cut-off edge for each cytokine. The use of such a global median cut-off for each cytokine allows for multiple comparative analyses between groups using the same criteria. Following data categorization, gray-scale diagrams were assembled to compile the cytokine pattern (columns) for each volunteer (rows). Column statistics was run for each diagram to calculate the frequency of “High Cytokine Producers” for each subject group. Next, the frequencies of “High Cytokine Producers” were ordered in an ascendant manner to create the ascendant curves referred to as “cytokine signatures.” From each cytokine signature curve, the attributes with frequencies greater than 50% were selected for profile analysis. Then, cytokine ratios were calculated to evaluate the immunological profile for each subject group. The non-parametric Mann–Whitney *U* test was used to assess significant differences. A *p* value < 0.05 was considered as statistically significant.

Another innovative strategy adopted was the inflammatory score, calculated according to previous studies (Duncan et al., 2003; Recasens et al., 2005; Biagi et al., 2010). Specifically, the inflammatory score was calculated based on three inflammatory cytokines, whose production was assessed as relevant in the HBP group according to the cytokine ratio (Figure 3) and signature analysis (Figure 4). To obtain such a score, the global median for each selected cytokine was calculated, and every value greater than the median gave 1 point to the subject. The inflammatory score ranged from 0 to 3. Scores 0 and 1 were categorized as “Not Inflamed” and scores 2 and 3 were categorized as “Inflamed.” Finally, the frequency of inflammatory score category for each group was calculated.

## Results

### Study Population

Eighty subjects (21 males and 59 females) aged 26–87 years (mean 64.5), were recruited for the present study. This population was stratified according to the presence of reported HBP (higher than 140 mmHg for systolic and 90 mmHg for diastolic) (Sociedade Brasileira de Cardiologia/Sociedade Brasileira de Hipertensão/Sociedade Brasileira de Nefrologia, 2010) and the chronic use of antihypertensive medication that kept their HBP under control. Forty-eight subjects had HBP (HBP group) while 32 had not (control). The demographic data of individuals are summarized in Table 1. No differences were found in age or gender distribution between the two groups.

**TABLE 1:** Demographic and health data of individuals from Casa Unimed in Governador Valadares, stratified by the presence of high blood pressure (HBP).

**Data**	**All subjects**	**Control group**	**HBP group**	***p*-value**
N.	80	32	48	–
Male	21	7	14	0.6056*
Female	59	25	34	
Age (mean ± SD)	64.5 ± 15.3	63.3 ± 15.0	65.3 ± 15.5	0.5611**
Age range	26–87	28–86	26–87	
High Blood Pressure	48	0	48	–

*, Fisher’s exact test; **, Student’s *t* test.

### Intestinal Microbiota Diversity Is Altered in Hypertensive Subjects

**FIGURE 1:**
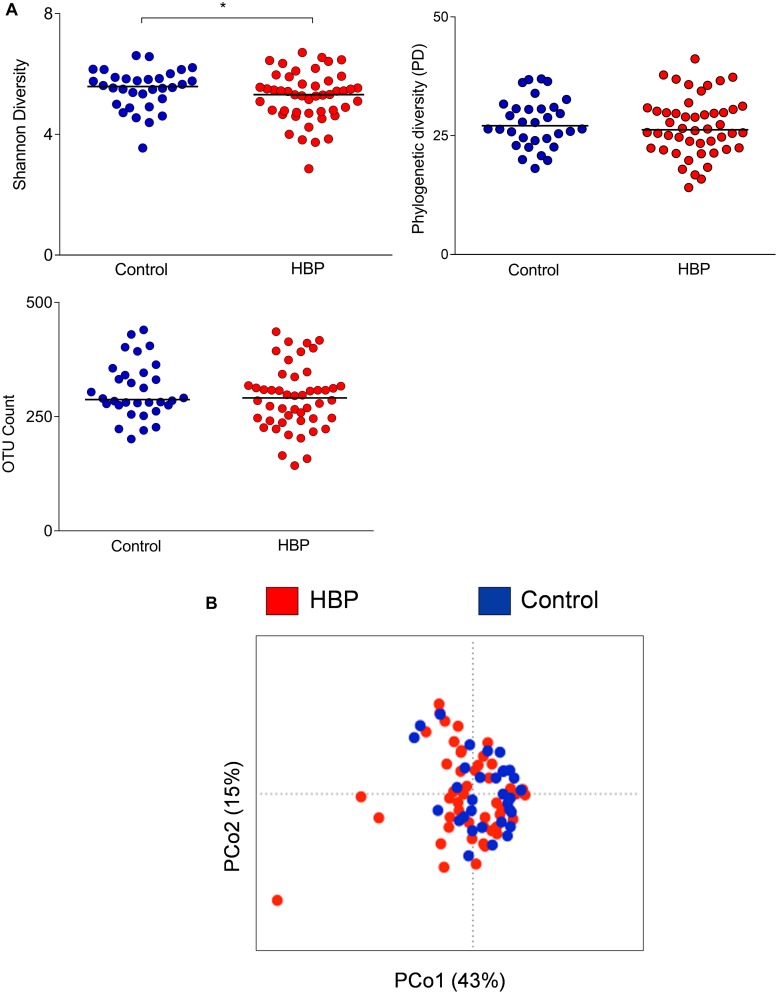
Comparison of the gut microbiota structure between hypertensive (HBP) and normotensive (control) subjects. **(A)** Alpha-diversity values were calculated according to the following metrics: the Shannon index for biodiversity, phylogenetic diversity (PD) and OTU count. *, *p* = 0.04, Mann–Whitney *U* test. **(B)** Principal Coordinates Analysis of the weighted UniFrac distances for HBP and control subjects. The two components explain 43 and 15% of the variance, respectively. A significant separation was found between the two groups (*p* = 0.04, permutation test with pseudo-F ratios). Red, HBP subjects (*n* = 48); blue, controls (*n* = 32).

Fecal samples were collected from the 80 subjects recruited in Governador Valadares, and the V3-V4 region of the bacterial 16S rDNA was sequenced. A total of 3,790,002 high-quality reads (mean per subject, 47,375; range, 3,745–1,50,790) were obtained and analyzed. Reads were clustered into 24,575 OTUs at 97% identity. Rarefaction curves obtained with phylogenetic diversity and OTU count did not reach saturation while the Shannon index for biodiversity plateaued at a sequence depth of about 1000 read counts (Supplementary Figure S1). According to this index (a combined parameter of richness and evenness), alpha diversity values were lower in the HBP group compared to normotensive controls (*p* = 0.04, Mann–Whitney *U* test; Figure 1A). Furthermore, HBP and control groups significantly, even if only barely, segregated according to weighted UniFrac distances (*p* = 0.04, *R*^2^ = 0.02, permutation test with pseudo-F ratios; Figure 1B). Specifically, the PCo1 axis, which accounted for 43% of the total variance in the dataset, was negatively associated with the presence of HBP (*p* = 0.03, Mann–Whitney *U* test).

### Hypertensive Subjects Show a Gut Microbiota Dysbiosis

At phylum level, the HBP-related gut microbiota tended to show a contraction of Bacteroidetes (mean relative abundance, 4.96% vs. controls, 8.97%; *p* = 0.03, FDR-adjusted *p* = 0.1, Mann–Whitney *U* test), resulting in a significantly increased Firmicutes/Bacteroidetes ratio in hypertensive vs. normotensive subjects (*p* = 0.03) (Supplementary Figure S2).

**FIGURE 2:**
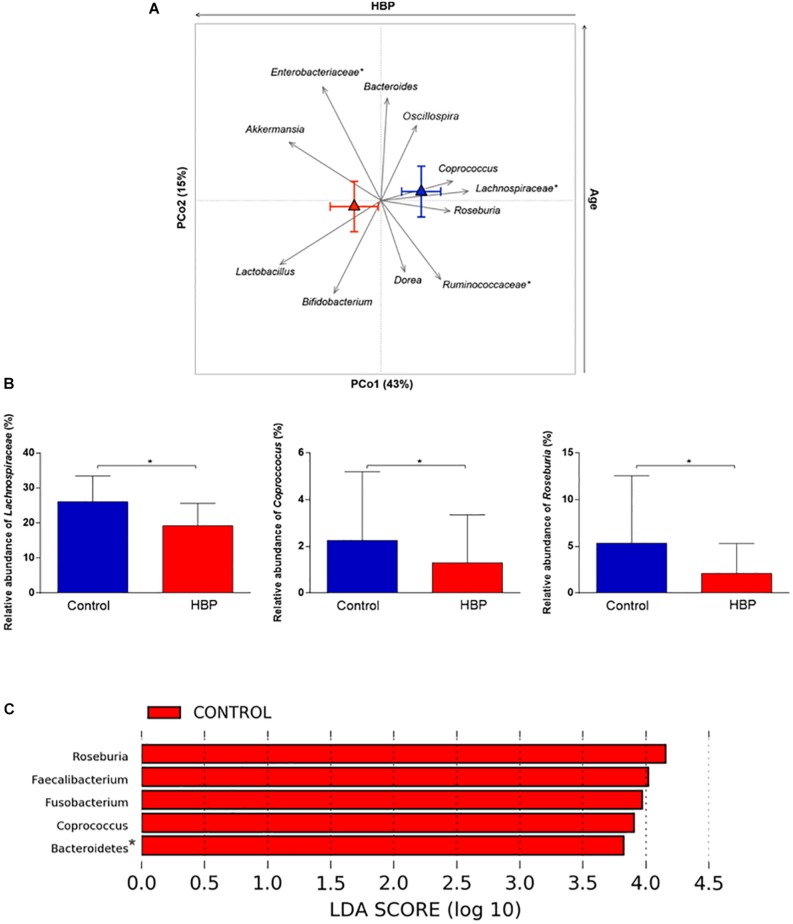
Intestinal microbial components driving the separation between hypertensive (HBP) and normotensive (control) subjects. **(A)** Genus-level relative abundance vectors with statistically significant contribution to the ordination space (*p* < 0.05, permutational correlation test) were overlaid onto the PCoA plot of weighted UniFrac distances (see Figure 1B). Triangles, centroids for each group (red, HBP; blue, control) with indication of standard errors on each coordinate axis. *, unclassified OTU reported at higher taxonomic level. PCo1 was negatively associated with the presence of HBP while PCo2 correlated positively with age. **(B)** Bar plots showing the relative abundance of the *Lachnospiraceae* family and the genera *Coprococcus* and *Roseburia* in HBP and control subjects. *p = 0.02; *p* = 0.04; *p* = 0.003, respectively; Mann–Whitney *U* test. **(C)** Linear discriminant analysis (LDA) effect size (LEfSe) analysis. LDA scores indicate differentially represented genera between groups (the logarithmic threshold for discriminative features was set to 2.0). *, unclassified OTU reported at higher taxonomic level.

In order to identify the bacterial genera responsible for the separation between the microbiota structure of HBP and control subjects, relative abundance vectors with a statistically significant contribution were identified and overlaid onto the ordination space based on weighted UniFrac distances of Figure 1B (*p* < 0.05, permutational correlation test). Interestingly, several microbial taxa were found to drive the clustering pattern, i.e., *Lactobacillus*, *Akkermansia*, *Bifidobacterium*, and unclassified *Enterobacteriaceae*, which were associated with HBP, and well-known butyrate producers of the *Lachnospiraceae* and *Ruminococcaceae* families, including *Roseburia*, *Coprococcus*, *Dorea*, and *Oscillospira*, which were associated with controls (Figures 2A,B). Most of these genera were also identified as discriminating according to LEfSe analysis (Figure 2C). These differences are likely to be attributable to the species *Roseburia faecis*, *Faecalibacterium prausnitzii*, *Parabacteroides distasonis*, and unclassified species belonging to the genera *Fusobacterium* and *Coprobacillus*, which were representative of normotensive controls, and *Lactobacillus salivarius*, *Bacteroides plebeius* and an unclassified species belonging to *Eggerthella*, which were characteristic of hypertensive individuals (*p* ≤ 0.05, FDR-adjusted *p* ≤ 0.1, Mann–Whitney *U* test) (Supplementary Figure S3). According to BLAST, *Eggerthella*, *Fusobacterium* and *Coprobacillus* OTUs showed the highest percent identity with *Eggerthella lenta* (91%), *Fusobacterium (pseudo)periodonticum* (99%), and *Coprobacillus cateniformis* (95%), respectively. However, it should be stressed that, given the known resolution limits of 16S rRNA gene-based sequencing, these species-level results must be interpreted with extreme caution, at least until further information, through other approaches possibly based on full-length 16S rRNA gene sequencing or shotgun metagenomics, is available.

### The Gut Microbiota Dysbiosis in Hypertensive Subjects Is Likely Independent of Age and Macronutrient Intake

Given the wide age range of volunteers, spanning young adults and elderly, we investigated the impact of age on the gut microbiota composition in our study cohort. According to weighted UniFrac distances, age was not associated with PCo1 (i.e., the axis along which HBP and control subjects clustered significantly), suggesting that the intestinal microbial dysbiosis in HBP may be independent of it (see Supplementary Figure S4). It was instead shown to correlate with the second axis of the PCoA plot, parallel to the relative abundance of several bacterial taxa (Figure 2A), confirming previous (HBP-independent) observations on the dynamics of the gut microbiota during aging (Odamaki et al., 2016; Biagi et al., 2017). In particular, higher age was found to be associated with higher proportions of *Oscillospira* and especially *Enterobacteriaceae*, already known to increase in the elderly in a sort of self-sustained pro-inflammatory loop. On the other hand, as expected, lower age was associated with increasing amounts of health-promoting bifidobacteria, along with SCFA producers of the gut microbiota, in particular *Dorea* and *Ruminococcaceae* members.

Furthermore, given the pivotal role of diet in shaping the gut microbiota (Zmora et al., 2019), we evaluated the dietary habits of volunteers through 3-day food frequency recalls. According to our data, no differences in the proportion of macronutrients between HBP and control individuals were observed (see Supplementary Table S1).

### Hypertensive Subjects Show an Overall Inflammatory Cytokine Profile

**FIGURE 3:**
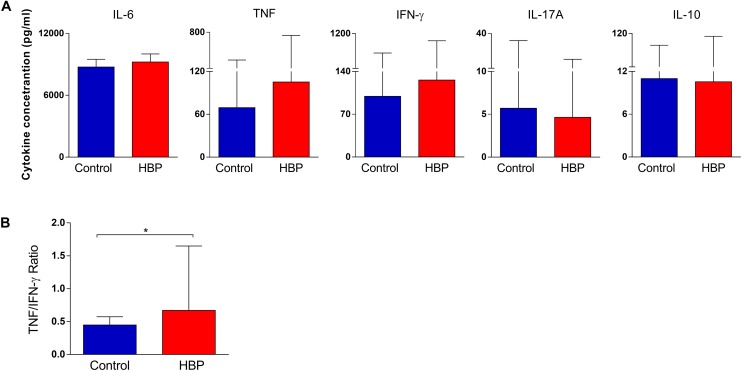
Comparison of cytokine production in the supernatant of PBMCs between the high blood pressure group (HBP) and control group (Control). **(A)** The bars represent the median and interquartile of the cytokine concentration (pg/ml), as determined by flow cytometry, for each study group. **(B)** The bars represent the TNF/IFN-γ ratio. *, *p* = 0.02, Mann–Whitney *U* test.

In order to assess whether the hypertensive subjects also had a different immunological profile compared to controls, the cytometric bead array (CBA) method was used to evaluate the production of IL-6, IFN-γ, TNF-α, IL-17A, and IL-10. We excluded IL-4 and IL-2 from the analysis, because their measurements were too low (median = 0 for both cytokines). According to our findings, there were no differences in the cytokine concentration between control and HBP groups (*p* > 0.05, Mann–Whitney *U* test; Figure 3A). However, to fully explore the possible contribution of these cytokines to the immunological profile of our study groups, we calculated several cytokine ratios, commonly used in immunological analysis (Gómez et al., 2004; Coelho-dos-Reis et al., 2013; Tsurumi et al., 2016; Medeiros et al., 2018), by creating a measure of balance between cytokine profiles. Interestingly, an increased TNF-α/IFN-γ ratio was found in HBP compared to the control group (*p* = 0.02, Mann–Whitney *U* test; Figure 3B), suggesting an overall inflammatory profile for HBP subjects. No difference was detected for the other ratios (data not shown).

**FIGURE 4:**
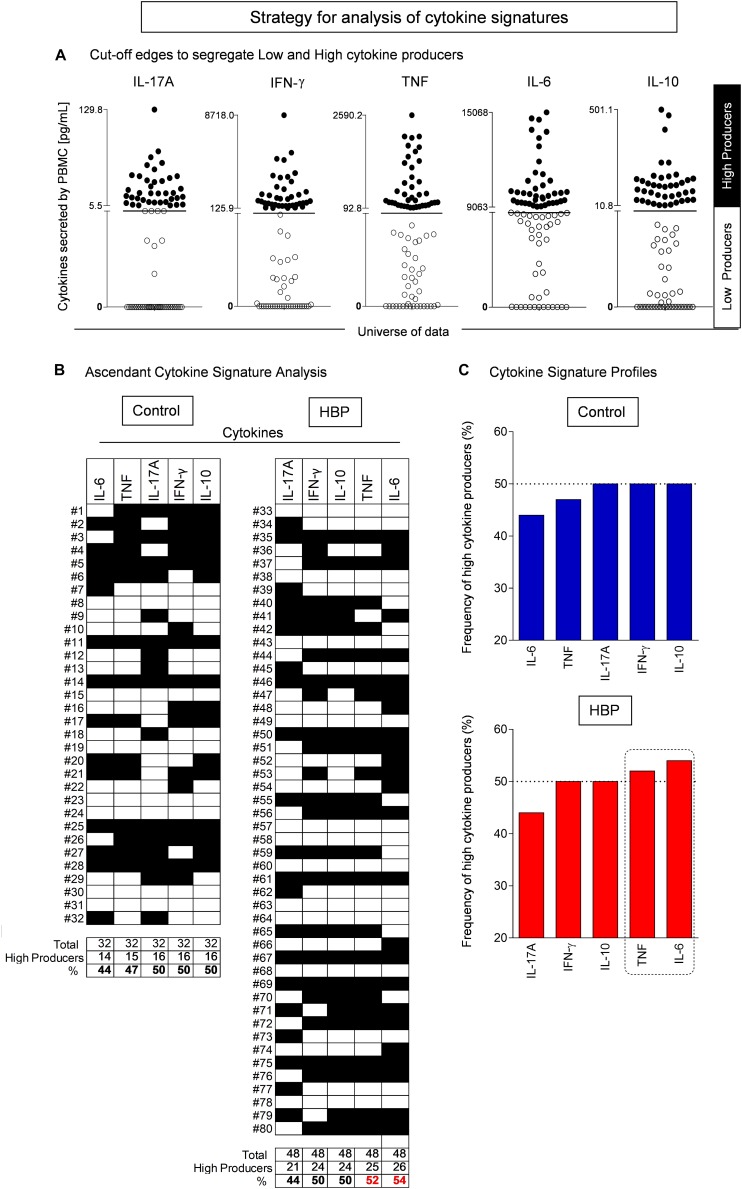
Cytokine signature analysis in hypertensive (HBP) and normotensive (control) individuals. **(A)** Establishing cut-off edges to categorize the subjects as “Low” or “High” cytokine producers. The cytokine quantitative analysis was performed by flow cytometry using the Cytometric Bead Array as described in Methods. The cut-off line was established as the global median value calculated for each cytokine, taking the whole dataset, including all subjects. The use of such a global median cut-off for each cytokine subsidizes multiple comparative analyses between groups using the same criterion. The subjects were then categorized as “Low” (white box) or “High” (black box) cytokine producers. **(B)** Assembling the ascendant cytokine signatures. Diagrams were assembled to compile the cytokine pattern (columns) for each volunteer (lines). Column statistics was run for each diagram to calculate the frequency of “High Cytokine Producers” for each group of patients. **(C)** The cytokine signature profiles. The frequencies of “High Cytokine Producers” were ordered in an ascendant manner to create the ascendant patterns referred to as “cytokine signatures.” From each cytokine signature curve, the attributes with frequencies higher than 50% (i.e., more than half of the group are high cytokine producers) were selected (dashed rectangle).

To further deepen the contribution of these cytokines, individuals were stratified in carriers of either low or high cytokine producers taking the global median of the cytokine index as the cut-off (Figure 4; please see the section “Materials and Methods” for the functional cytokine signature analysis), as previously reported (Luiza-Silva et al., 2011; Campi-Azevedo et al., 2012; Silveira-Nunes et al., 2017). When considering the frequency of high cytokine producers, a relevant production (i.e., more than 50% of individuals were high producers) was only found in the HBP group for TNF-α and IL-6 (Figure 4B).

**FIGURE 5:**
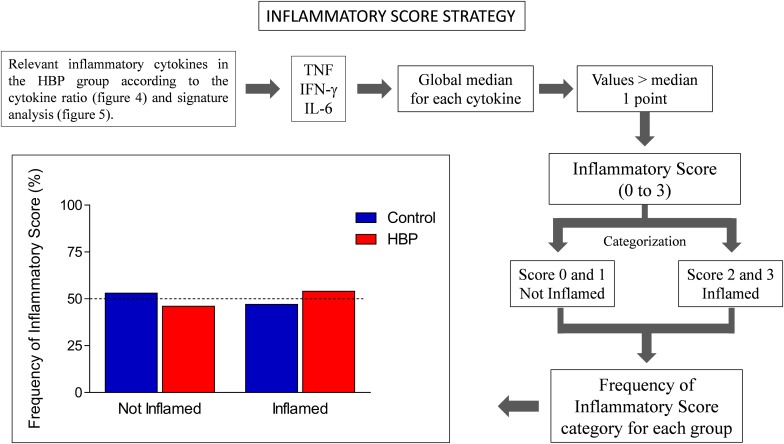
Inflammatory score in hypertensive (HBP) and normotensive (control) subjects. The inflammatory score was calculated based on three inflammatory cytokines, whose production was assessed as relevant in the HBP group according to the cytokine ratio and signature analysis, i.e., IFN-γ, TNF, and IL-6. The global median for each selected cytokine was calculated, and every value greater than the median gave 1 point to the subject. The sum of the points for each subject defined its Inflammatory Score. The inflammatory score ranged from 0 to 3. Scores 0 and 1 were categorized as Not Inflamed and scores 2 and 3 were categorized as Inflamed. The frequency of inflammatory score category for each group was calculated. The bars represent the frequency of Inflamed (score 2–3) and Not Inflamed (score 0–1) subjects in control (blue) and HBP group (red).

Finally, to support the hypothesis of increased inflammation in HBP subjects, an inflammatory score, based on three inflammatory cytokines, whose production was assessed as relevant in the HBP group according to the cytokine ratio and signature analysis, i.e., IFN-γ, TNF-α, and IL-6 (please see section “Materials and Methods”), was calculated. Subjects were considered as inflamed for inflammatory scores of 2 and 3, and as non-inflamed for scores of 0 or 1. In this analysis (Figure 5), we observed that more than half (50% as a cut-off) of the individuals from the HBP group (54%) were categorized as inflamed by inflammatory score, a result we did not observe in the control group (47%).

## Discussion

Hypertension is a multifactorial clinical condition characterized by high and sustained levels of blood pressure, likely resulting from a complex interplay of endogenous and environmental factors, including the gut microbiota (Mell et al., 2015; Moghadamrad et al., 2015; Yang et al., 2015; Durgan et al., 2016; Galla et al., 2017; Oyama and Node, 2019). Epidemiological studies have shown that high levels of blood pressure are widely associated with increased risk of fatal and non-fatal cardiovascular events (Lewington et al., 2002; Williams et al., 2008). Although research in the field has grown considerably in recent years, the etiology of hypertension is poorly understood and there is still limited evidence on the role of the gut microbiota.

In an attempt to bridge these gaps, here we sequenced the bacterial 16S rRNA gene of fecal samples from a cohort of 48 hypertensive and 32 normotensive Brazilian individuals, and integrated the analysis with extensive immunological profiling. Consistent with recent findings in Chinese and United States cohorts (Li et al., 2017, 2019; Yan et al., 2017; Dan et al., 2019; Sun et al., 2019), our study highlighted a dysbiosis of the intestinal microbiota in hypertensive subjects, featured by reduced biodiversity and distinct bacterial signatures compared with the normotensive counterpart (see Supplementary Table S2 for a review of available studies on gut microbiota and hypertension). Loss of microbial diversity is widely recognized as a hallmark of unhealthy microbiota and so far has been reported in a plethora of human disorders, including (but not limited to) gastrointestinal, immune and metabolic ones (Sonnenburg and Sonnenburg, 2014), thus likely to be part of a non-specific, shared response to the disease. With regard to the taxonomic structure, we found a higher Firmicutes/Bacteroidetes (F/B) ratio in the gut microbiota of hypertensive individuals compared to controls, resulting from a reduction in the relative proportion of Bacteroidetes. Interestingly, also Yang et al. (2015) reported an increased F/B ratio in a small group of hypertensive patients, as well as in rat models of hypertension. Although still debated, the F/B ratio has been widely used in the past as a marker of an obesogenic microbiota, with high values being generally associated with the consumption of high-fat low-fiber Western diets and inflammation (Turnbaugh et al., 2009; Everard and Cani, 2013; Trompette et al., 2014; Nakayama et al., 2017).

At the genus level, our results confirmed previous evidence on the relative abundance decrease of several SCFA (mainly butyrate) producers, generally recognized as beneficial components of the human gut microbiota, including members of the *Lachnospiraceae* and *Ruminococcaceae* families, such as *Roseburia* and *Faecalibacterium* (Li et al., 2017, 2019; Yan et al., 2017; Dan et al., 2019). Such alterations could therefore represent robust dysbiotic microbial signatures, regardless of geography and lifestyle. SCFAs are known to exert a key, multifactorial role in human physiology, not only locally but also at distant body sites, promoting the maintenance of metabolic and immunological homeostasis (Koh et al., 2016). In particular, they have been shown to regulate blood pressure via the sensory receptors Olfr78 (Olfactory receptor 78) and GPR41 (G protein-coupled receptor 41), acting in opposition to one another (Pluznick, 2014), and exert relaxant effects on resistance arteries (Mortensen et al., 1990), thus possibly improving microcirculation. Furthermore, SCFAs are known to have important anti-inflammatory effects (Vinolo et al., 2011; Vieira et al., 2012; Chang et al., 2014; Iraporda et al., 2015; Park et al., 2015; Yang and Zubcevic, 2017). Specifically, they are able to inhibit the activation of NF-κB in immune cells by binding to GPR43 and GPR41, thereby blocking inflammatory responses and suppressing the production of TNF-α and IL-6. Butyrate also suppresses inflammation by reducing IL-12 and increasing IL-10 expression (Säemann et al., 2000; Fukae et al., 2005; Tedelind et al., 2007), and it promotes transcription of the *Foxp3* gene, inducing the production of regulatory T cells (Furusawa et al., 2013). As thoroughly discussed by Million et al. (2018), butyrate could represent the best functional marker of the healthy mature anaerobic gut microbiota, and oxidative stress-sensitive butyrate-producing commensals could serve as new instrumental targets for the maintenance of a microbiota-immunity symbiotic loop and the prevention of a series of local and systemic dysbiosis-related disorders. It is also worth noting that *Roseburia*, one of the main butyrate-producing microbes that were found to be underrepresented in hypertension, also produces conjugated linoleic acid, which has been shown to have anti-inflammatory properties and potential blood pressure-lowering effects as well (McIntosh et al., 2009; Neyrinck et al., 2012; Van den Abbeele et al., 2013; Derrien and Veiga, 2017).

Interestingly, our study also led to the identification of other bacteria deserving further investigation for their possible role in hypertension, especially *Lactobacillus* (most likely *L. salivarius*), *Eggerthella* and possibly *B. plebeius*, which were found to be discriminating for hypertensive individuals. While the literature is consistent in reporting both *Eggerthella* and *B. plebeius* as potential pathogens for cardiovascular disease, with the former already found to occur at higher levels in patients with hypertension compared to controls (Yan et al., 2017) and the latter being associated with dyslipidemia (Liu et al., 2019), conflicting evidence is available for lactobacilli. Some clinical trials in fact support hypotensive effects for *Lactobacillus* species used as probiotics (Upadrasta and Madempudi, 2016), but this genus and/or certain species have also been found to be overabundant in some inflammatory disorders, including obesity (Million et al., 2012), coronary heart disease (Yamashita, 2017), and heart failure (Kamo et al., 2017). In particular, the species *L. salivarius* has recently been assigned to metagenomics linkage groups enriched in the gut microbiome of individuals with atherosclerotic cardiovascular disease (Jie et al., 2017), thus opening fascinating perspectives on the possible use of this species as a non-invasive biomarker for blood pressure-related diseases. It is also worth noting that we found an association between hypertension and the mucin degrader *Akkermansia*. Although this microorganism is generally associated with improved metabolic profile (Everard et al., 2013), it has recently been reported to show greater abundance in hypertensive Chinese subjects (Dan et al., 2019) and exacerbate inflammation during infections by disturbing the mucus layer homeostasis (Ganesh B. P. et al., 2013). Recent evidence in human PBMCs and mice also indicates that *Akkermansia* promotes Th1 lymphocyte differentiation (Cekanaviciute et al., 2017). It is thus tempting to speculate that this microbiota member may play a role in hypertension, by sustaining and contributing to an overall pro-inflammatory environment.

Consistent with our hypothesis, we observed an inflamed immune profile in hypertensive individuals. Chronic inflammation is one of the biological mechanisms most commonly associated with hypertension (Blake et al., 2003; Cevenini et al., 2010; Chung et al., 2010; Singh and Newman, 2011; Youn et al., 2013; De Miguel et al., 2015). Bautista et al. (2005) demonstrated that the plasma levels of inflammatory cytokines, such as C-reactive protein, IL-6, and TNF-α, positively correlated with blood pressure in humans. In agreement with those findings, our results revealed an increase of type 1 cytokines, with an increased TNF/IFN-γ ratio, in hypertensive individuals when compared to normotensive ones, thus strengthening the hypothesis that inflammation plays an important role in hypertension. Specifically, according to our ascending profile analysis, TNF and IL-6 were the main cytokines responsible for the leukocyte immune profile of hypertensive individuals, and 50% of them had high inflammatory scores. The association of elevated levels of TNF-α with hypertension has been demonstrated through pharmacological and genetic approaches in experimental models, including angiotensin II-induced hypertension, lupus, metabolic syndrome and preeclampsia (Zinman et al., 1999; Ito et al., 2001; Guzik et al., 2007; Venegas-Pont et al., 2010; Ramseyer and Garvin, 2013). The IL-6 levels are also frequently elevated under hypertensive conditions. In particular, studies have shown that IL-6 is essential for the development of angiotensin II-induced hypertension and that the activation of the STAT3/JAK pathway by IL-6 plays a key role in the disease (Brands et al., 2010). In addition, human studies confirmed increased plasma levels of IL-6 in response to acute angiotensin II infusion, and also in hypertensive patients (Furuya et al., 2010; Chamarthi et al., 2011; De Miguel et al., 2015).

In summary, despite the small sample size, our work provides, to the best of our knowledge, the first evidence of an association of hypertension with altered gut microbiota and inflammation in a Brazilian population. While lending support to the existence of microbial signatures of hypertension, possibly robust to age and geography, and stressing the need for species-level analysis in future microbiome-based studies, our findings point to bacteria widely neglected to date as potential contributors to the loss of intestinal homeostasis, and emphasize the high vulnerability of hypertensive individuals to inflammation-related disorders. Future studies in independent and much larger cohorts, also through other methods, including culture-dependent ones, are encouraged to assess alterations in the microbiota-host co-metabolic networks and their contribution to hypertension and related complications. This information will be instrumental to the design of rational nutritional interventions aimed at correcting the gut dysbiosis and mitigating dysregulated immune responses, for reduced incidence and severity of chronic diseases.

## Data Availability Statement

The 16S rRNA gene sequences generated for this study can be found in the MG-RAST database (project ID, mgp84730).

## Ethics Statement

The studies involving human participants were reviewed and approved by the Ethical Committee of Universidade Federal de Minas Gerais (UFMG) as well as the National Research Ethics Committee (CONEP) of Brazil. The patients/participants provided their written informed consent to participate in this study.

## Author Contributions

AF and PB conceived and designed the study. LA and EC recruited volunteers, collected biologic samples, and applied the questionnaires. DD, AV, and GS-N performed the microbial DNA extraction from feces. GS-N and ST performed the 16S rRNA gene sequencing. GS-N, ST, and SR carried out the bioinformatics analysis and analyzed the microbiota data. TM and EC collected and analyzed the nutritional data. GS-N, ES, OM-F, RC-O, and AT-C performed and reviewed the cytokine measurements. GS-N and SR prepared Figures 1–3 and Supplementary Figure S1. GS-N and ST wrote the main manuscript text. AF, OM-F, and RC-O revised and edited the draft. All authors read and approved the final manuscript.

## Conflict of Interest

The authors declare that the research was conducted in the absence of any commercial or financial relationships that could be construed as a potential conflict of interest.
