# Supplementary material for: Hypertension Is Associated With Intestinal Microbiota Dysbiosis and Inflammation in a Brazilian Population
Source: Front Pharmacol. 2020 Mar 12;11:258. doi: 10.3389/fphar.2020.00258 (PMC7080704; doi:10.3389/fphar.2020.00258)
Supplement: Supplementary file 1 [file Data_Sheet_1.PDF]

Supplementary Material

Supplementary Figures

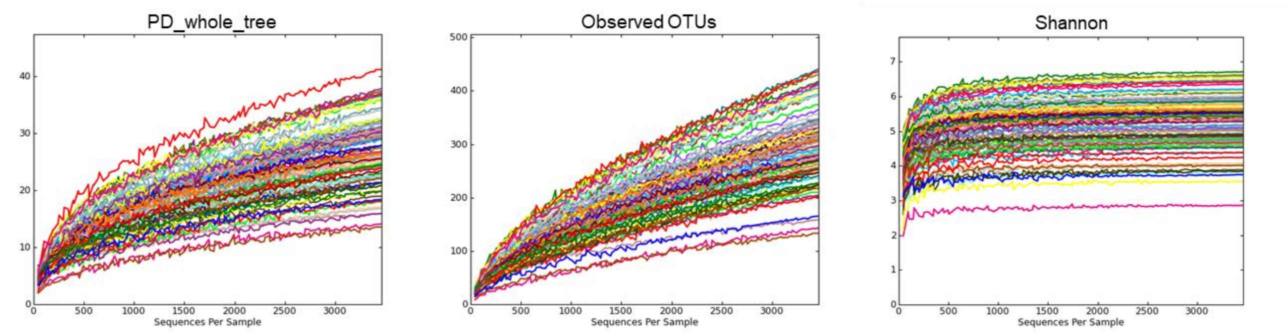

Supplementary Figure 1. Rarefaction curves. The following metrics were used: phylogenetic diversity (PD_whole_tree), the number of observed OTUs and the Shannon index. Curves are color-coded by subject.

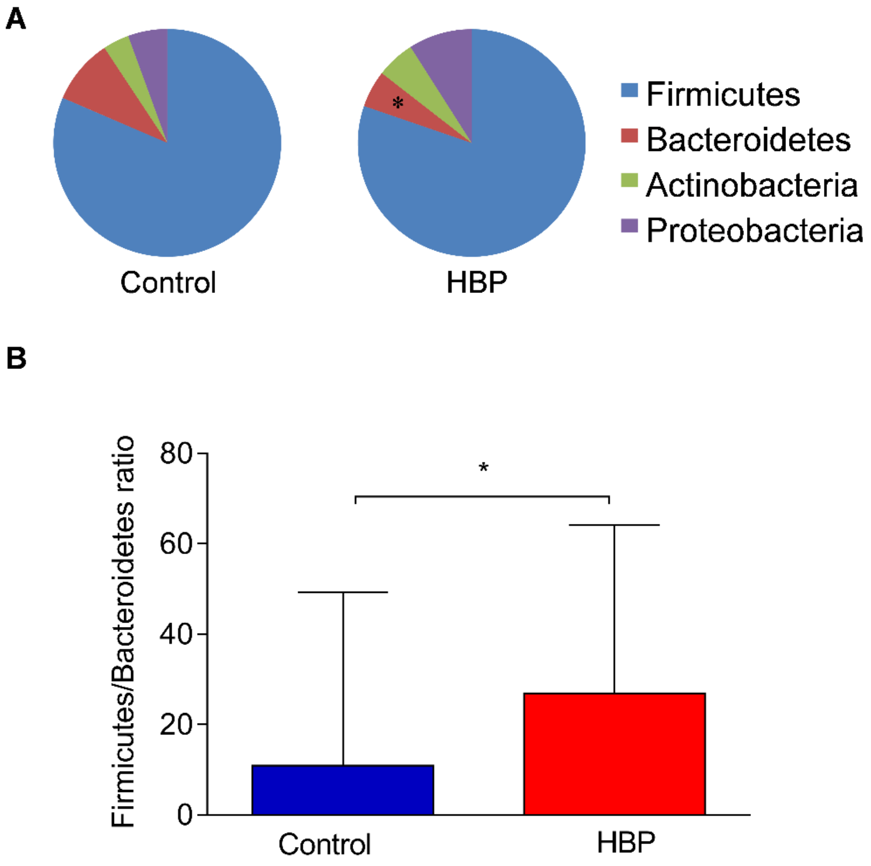

Supplementary Figure 2. Phylum-level gut microbiota composition in hypertensive (HBP) and normotensive (control) subjects. A. Pie charts summarizing the relative abundance of the four most abundant phyla in HBP subjects and controls. **B.** Firmicutes/Bacteroidetes ratio. *, p=0.03, Mann-Whitney U test.

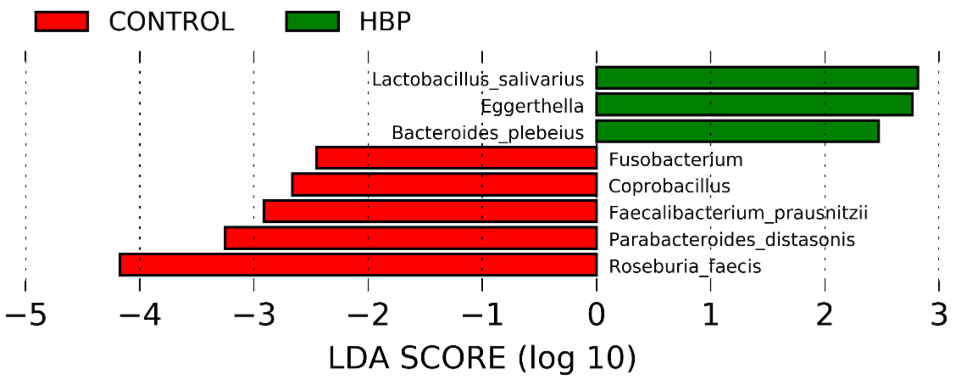

Supplementary Figure 3. Intestinal microbiota species potentially driving the separation between hypertensive (HBP) and normotensive (control) subjects. Linear discriminant analysis (LDA) effect size (LEfSe) analysis. LDA scores indicate differentially represented species between groups (the logarithmic threshold for discriminative features was set to 2.0). For *Eggerthella*, *Fusobacterium* and *Coprobacillus*, the species were unclassified.

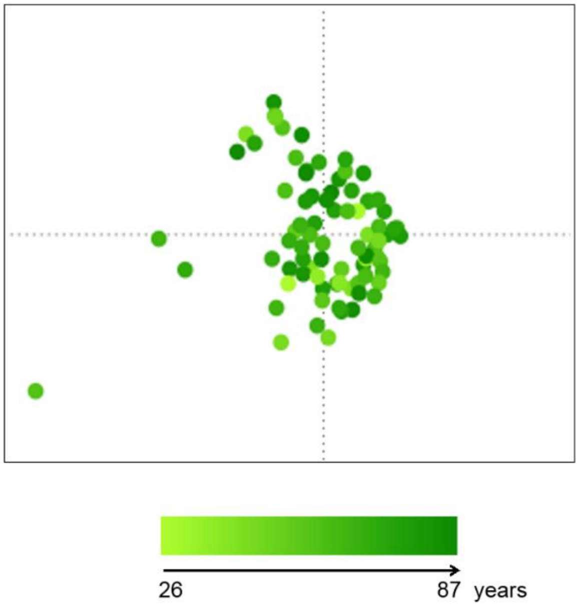

Supplementary Figure 4. The gut microbiota dysbiosis in a cohort of Brazilian hypertensive subjects is independent of age. Principal Coordinates Analysis of the weighted UniFrac distances for hypertensive and control subjects. Dots are colored by subject age (see the color gradient at the bottom). See also Figure 1B.

Supplementary Tables

Supplementary Table 1. Frequency of macronutrients intake in high blood pressure group (HBP) and control group (Control).

	Macronutrient		
	Protein	Fat	Carbohydrate
Total	19%	28%	53%
Control	17%	26%	57%
HBP	19%	30%	51%

Supplementary Table 2. Summary of published studies on human gut microbiota and hypertension. Only research articles based on next-generation sequencing technologies were considered. Genus-level differences between hypertensive (case) and normotensive (control) subjects or associations with blood pressure are reported. Results consistent with our study are highlighted in bold.

Number of subjects and characteristics[a]	Geographical origin	Positively associated with or increased in relative abundance in hypertension	Negatively associated with or decreased in relative abundance in hypertension	Ref.
99 cases (53.6 yrs, 6.1%)<br>41 controls (53.7 yrs, 21.9%)	Tangshan, China	*Prevotella*, *Klebsiella*, *Porphyromonas*, and *Actinomyces*	***Faecalibacterium***, ***Bacteroides***, *Oscillibacter*, ***Roseburia***, *Bifidobacterium*, ***Coprococcus***, and *Butyrivibrio*	Li et al., 2017
60 cases (57.0 yrs, 41.7%)<br>60 controls (56.0 yrs, 46.7%)	Harbin, China	*Klebsiella*, *Clostridium*, *Streptococcus*, *Parabacteroides*, ***Eggerthella***, *Salmonella*, ***Bacteroides***, *Sutterella*, and *Pyramidobacter*	***Faecalibacterium***, ***Roseburia***, *Synergistetes*, ***Bacteroides***, *Megasphaera*, and *Aeromicrobium*	Yan et al., 2017
22 cases (NA)<br>18 controls (NA)	Florida, US	*Alistipes*, *Ruminococcus*, and *Eubacterium*	***Bacteroides***	Kim et al., 2018
62 cases (69.3 yrs, 53.2%)<br>67 controls (69.5 yrs, 61.0%)	Beijing, China	*Alistipes*, ***Bacteroides***, *Barnesiella*, *Butyricimonas*, *Christensenella*, *Clostridium sensu stricto*, *Cosenzaea*, *Desulfovibrio*, *Dialister*, *Eisenbergiella*, *Faecalitalea*, *Megasphaera*, *Microvirgula*, *Mitsuokella*, *Parabacteroides*, *Proteiniborus*, and *Terrisporobacter*	*Acidaminobacter*, *Adlercreutzia*, *Anaerotruncus*, *Asteroleplasma*, *Bulleidia*, *Cellulosilyticum*, *Clostridium III*, ***Clostridium IV***, ***Clostridium XlVa***, *Coprobacter*, *Enterococcus*, *Enterorhabdus*, *Flavonifractor*, *Gemmiger*, *Guggenheimella*, *Intestinimonas*, ***Lachnospiracea_incertae_sedis***, *Lactivibrio*, *Lactobacillus*, *Macellibacteroides*, *Marvinbryantia*, *Olsenella*, *Paraprevotella*, *Parasutterella*, *Phascolarctobacterium*, *Prevotella*, *Romboutsia*, *Ruminococcus*,	Dan et al., 2019

			Sporobacter, *Sporobacterium*, *Sutterella*, *Vampirovibrio*, *Veillonella*, and *Victivallis*	
38 cases (52.5 yrs, 0%)<br>9 controls (46.2 yrs, 0%)	Liège, Belgium	*Clostridium sensu stricto*	***Ruminococcaceae****, and *Clostridiales**	Huart et al., 2019
63 cases (58.4 yrs, 44.4%)<br>42 controls (59.3 yrs, 59.5%)	Xinxiang county, China	*Tyzzerella*, *Enterobacter*, *Actinobacillus*, *Sutterella*, *Parasutterella*, *Paenibacillus*, *Prevotella*, *Vibrio*, *Holdemanella*, *Dongia*, *Romboutsia*, *Flavonifractor*, and *Glutamicibacter*	*Chitinibacter*, ***Bacteroides***, *Faecalitalea*, ***Dorea***, *Phascolarctobacterium*, *Pediococcus*, *Lachnospira*, *Megamonas*, ***Faecalibacterium***, ***Fusobacterium***, *Terrimonas*, and *Erysipelatoclostridium*	Li et al., 2019
20 cases (NA)<br>10 controls (NA)	Shaanxi, China	*Prevotella*, *Megasphaera*, *Parasutterella*, *Phascolarctobacterium*, and *Escherichia-Shigella*	***Faecalibacterium***	Mushtaq et al., 2019
186 cases (NA)<br>343 controls (NA)	Birmingham, Chicago, Minneapolis, Oakland, US	*Veillonella*, *Robinsoniella*, and *Catabacter*	*Akkermansia*, *Ruminococcus*, *Anaerovorax*, *Sporobacter*, *Asaccharobacter*	Sun et al., 2019

[a] Mean age and percentage of females are shown in brackets.
NA, not available.
*, unclassified OTU reported at higher taxonomic level.

Supplementary References

Huart, J., Leenders, J., Taminiau, B., Descy, J., Saint-Remy, A., Daube, G., et al. (2019). Gut microbiota and fecal levels of short-chain fatty acids differ upon 24-hour blood pressure levels in men. *Hypertension* 74(4), 1005-1013. doi: 10.1161/HYPERTENSIONAHA.118.12588

Kim, S., Goel, R., Kumar, A., Qi, Y., Lobaton, G., Hosaka, K., et al. (2018). Imbalance of gut microbiome and intestinal epithelial barrier dysfunction in patients with high blood pressure. *Clin. Sci. (Lond).* 132(6), 701-718.

Mushtaq, N., Hussain, S., Zhang, S., Yuan, L., Li, H., Ullah, S., et al. (2019). Molecular characterization of alterations in the intestinal microbiota of patients with grade 3 hypertension. *Int. J. Mol. Med.* 44(2), 513-522. doi: 10.3892/ijmm.2019.4235
